# Thinking about COVID-19 Scenario in Brazil: The Alternation between the Useful, the Uncertain and the Futile

**DOI:** 10.1055/s-0040-1717142

**Published:** 2020-09-29

**Authors:** Bruno Ramalho de Carvalho, Fernanda Fernandes Fonseca, Henrique de Barros Moreira Beltrão

**Affiliations:** 1 Hospital Sírio-Libanês, Brasília, Distrito Federal, Brazil; 2 Ministério da Saúde, Brasília, Distrito Federal, Brazil

The only pandemic comparable to the current event caused by the new coronavirus (SARS-CoV-2), the disease called COVID-19, was that of the Spanish Flu in 1918. 1 At that time, with slow and scarce intercontinental transport, diseases spreading infectious diseases were unlikely. Nowadays, on the contrary, the ability to move a highly infectious virus is enormous. Likewise, information, whether scientific or opinionated, moves easily around the world today. Not casually, the term “viralization” is used when any information quickly reverberates through the internet.

In this context, the useful, the uncertain and the futile alternate in published news about COVID-19. And the scientific literature is not shielded from this. It can be said that the disease caused by SARS-CoV-2 is for health research today as nothing seems to have been. It is a completely uncontrolled worldwide phenomenon. The scientific community inhales and exhales COVID-19 in 2020. The disease is a fever, not just literally, and we are still looking for a good way to fight it.

In addition to what we mentioned above, is the fact that, in some way, any researcher in the field, anywhere, wants to discover how to free the world from COVID-19 and raise the glories of a new discovery for themselves or their work group. To that end, the number of published studies and texts grows so quickly that it becomes almost impossible to follow a reliable line of reasoning or to envision a truth. For the reader to understand what it is about, querying the term COVID-19 to the PubMed database ( https://pubmed.ncbi.nlm.nih.gov ) on August 14, 2020 resulted in an impressive 40,850 references. A little more than 8 months ago, when the disease appeared, predicting a scientific production with such a volume in such a short time would sound absurd. Limiting ourselves to a comparison with a relatively recent example, the search for the term Zika in that database, on the same date, resulted in about 8 thousand and two hundred references, and 5 years have passed since the identification of its correlation with the epidemic of microcephaly among exposed fetuses. It is through this path that the uncertainties are broadly presented.

Indeed, the rapid dissemination of data on COVID-19 or any other disease with a similar impact would be highly welcome by the medical community, but not without the scrutiny of the scientific method and the minimum time required for research. In the absence of that time and effective scrutiny tools, distorted versions of the facts easily intertwine with the relevant data and appropriate their reliable appearances. This ultimately generates countless interpretations for each relevant aspect of the disease. Yes, it is the effect of the post-truth that also echoes in science, when reason and emotion are mixed, taking people to the extreme of faithfully believing in the data that meet their fears and desires.

As we have suggested, science needs time to be a real science, usually a long time. And it is exactly in a scenario of anguish and collective uncertainties that the scientific method should be followed strictly, with well-defined research steps, leading to clear and reproducible results. In the COVID-19 pandemic, it would be important for the information to be made public only after the rigorous follow-up of cases, minimizing the impact of fragile and contradictory information, which highlights the general insecurity scenario. It would be a movement contrary to what we are experiencing, in which the accelerated consumption of pseudo-scientific or pre-scientific information occurs both through traditional documentation vehicles and through social networks.

Not without reason, the whole world is watching closely the curves of COVID-19 in Brazil. Currently, the Brazilian epidemic is one of the fastest growing in the world 2 ; our numbers of new cases and deaths, since the beginning of the pandemic until now, are only below the numbers observed for the United States. The national epidemiological curve, which describes daily case reports, and which is influenced by the curves of large cities, suggests that we may be on a plateau without knowing whether it will precede the epidemic's shrinkage or end in a second wave of growth. This is because countless mathematical models have been made available, without any of them having stood out for their reliability until now, especially when applied to a country of continental dimensions such as Brazil, in which the process of internalization of the disease may be just beginning.

It is important that health authorities and managers are aligned with scientific evidence so that their leadership on the epidemic reinforces measures with a positive impact on the health of the population and minimizes potential deviations caused by the dissemination of misleading and potentially harmful information. It is not what has happened, apparently. On the contrary, we are supporting the recommendation for the use of certain drugs in the treatment of COVID-19, when scientific evidence with the greatest possible impact indicates their ineffectiveness. 3 4 5 The result? Mistakenly, many Brazilian citizens have been inspired by the intransigence coming from health authorities and managers, and by the aggressiveness of positions pro- or counter- any intervention, making them echo, in spite of what happens in academic circles.

In truth, the problems are not limited to those described above. In Brazil, there are those who claim that the seriousness of COVID-19 is not true, based on the comparison between the accumulated numbers of deaths in 2020 and 2019, in the same period. According to this analysis, the disease should not kill so much, since the number of deaths appears to be lower in 2020. But we consider this conclusion to be wrong since the data from the Ministry of Health's Hospitalization System are not yet consolidated and, even if were, the independence (fluctuation) of numbers from one year to another is an essential precept to interpret them. Think outside the box: even if the number of deaths in 2020 is, so far, lower than the number of deaths in the same period last year, that argument would not be valid. Otherwise, we would have to conclude that the COVID-19 epidemic brought gains to Brazil and to thank SARS-CoV-2 for having reduced the number of hospital admissions and deaths. That would be a huge folly.

The issue of numbers is much more complex than it appears to be. The quality of the counting of cases of a specific disease depends on an efficient epidemiological surveillance in all stages, namely: making the diagnosis; filling in the notification itself; systematization and computerization of data by local epidemiological surveillance services, at municipal and state levels; integration and, finally, accounting at the national level. Thus, one cannot fail to glimpse the negative impact of an epidemic at each point in the information generation process, leading to the possibility of error in the final count of cases of the disease in question. We will be close to the real numbers only in the medium to long term, when the worst scenario is expected to have passed.

It is also possible that, with the burden caused by the COVID-19 epidemic in Brazil, other diagnoses are being underreported. If we see, for example, the decrease in the diagnosis of flu syndrome by other respiratory viruses (which, in fact, is happening), we could assume that there is less circulation of seasonal respiratory viruses, while we have greater circulation of SARS-CoV-2. But we can also think that, as a direct consequence of the epidemic, less diagnostic tests are being carried out to identify other etiologic agents, with less notification and surveillance regarding other respiratory viruses.

It is important to comment, at this point, on the strategy of social distancing. It is well known that this intervention is far from being an isolated solution to the epidemic in a country with the size and intellectual and cultural diversity of Brazil. But which other strategy of similar scope would we have to overcome social distancing in efficiency at this time? Mathematical calculations indicate the effectiveness of the combination of early lockdown, measures of social distancing and personal protection, 6 and their effectiveness seems to have been demonstrated elsewhere in the world, of which New Zealand is perhaps the prime example. 7 Furthermore, relaxation experiences without clear and well-understood rules for reopening economic activities can be catastrophic, with an important increase in the number of cases. The same calculations that point to the efficiency of social distancing estimate that, in scenarios of high incidence of the disease and confinement lasting less than 45 days, any relaxation should lead to new waves of growth of the epidemic. 6

Undeniably, the available knowledge suggests that we will only have a reassuring perspective when there is at least one of the following situations: (i) an effective drug, which can appear at any time, but without strong candidates so far; (ii) vaccination immunization, which is perhaps the most feasible and close to occurring, although we still do not have final information on the efficacy of the vaccines under study and it is not possible to predict the ability to distribute the vaccine to the population in sufficient quantities and in a short time; or (iii) herd immunity.

In principle, the herd immunity required to contain COVID-19 has been estimated to be close to 70%. 8 However, recently published studies suggest that it may be between 20 9 and 43%, 10 taking into account variations in transmissibility between different groups of people within the same population. To see such an abbreviation for collective immunity would mean bringing the horizons of resumption of social life and the economy closer together, which undoubtedly suffered intensely from the global health emergency that we are experiencing in 2020. On the other hand, it is frightening to think about the number of deaths we owe arrive before collective immunity is reached, even at 20%, considering the prevalence of infection among Brazilians, which is still supposedly small. By the way, it is true that the prevalence of SARS-CoV-2 may be underestimated, since few asymptomatic people are tested. Thus, it is very difficult to fit the pieces of this puzzle called COVID-19.

We dare to end the reflection by touching on philosophical questions. Issues related to COVID-19 have long since seemed to move away from science: today, the COVID-19 pandemic seems to be more of an object of passion and passionate people who usually only see what is desirable to see. In other words, in passion, facts are subject to the imagination of those who see them through the lens of their own expectations. There is no logical reasoning that stands out and, therefore, it threatens scientific thinking so much. Health professionals need to be aware of such deviations, because although there is always the possibility to rectify them, there is not always enough time to reverse their consequences. If the best way to deal with all of this is still not defined by science, it will certainly not be defined on its absence.
